# Urate transporter inhibitor lesinurad is a selective peroxisome proliferator-activated receptor gamma modulator (sPPARγM) in vitro

**DOI:** 10.1038/s41598-018-31833-4

**Published:** 2018-09-10

**Authors:** Pascal Heitel, Leonie Gellrich, Jan Heering, Tamara Goebel, Astrid Kahnt, Ewgenij Proschak, Manfred Schubert-Zsilavecz, Daniel Merk

**Affiliations:** 1 Goethe University Frankfurt, Institute of Pharmaceutical Chemistry, Max-von-Laue-Str. 9, D-60438 Frankfurt, Germany; 2 Project Group Translational Medicine and Pharmacology TMP, Fraunhofer Institute for Molecular Biology and Applied Ecology IME, Theodor-Stern-Kai 7, D-60596 Frankfurt, Germany

## Abstract

Gout is the most common arthritic disease in human but was long neglected and therapeutic options are not satisfying. However, with the recent approval of the urate transporter inhibitor lesinurad, gout treatment has experienced a major innovation. Here we show that lesinurad possesses considerable modulatory potency on peroxisome proliferator-activated receptor γ (PPARγ). Since gout has a strong association with metabolic diseases such as type 2 diabetes, this side-activity appears as very valuable contributing factor to the clinical efficacy profile of lesinurad. Importantly, despite robustly activating PPARγ *in vitro*, lesinurad lacked adipogenic activity, which seems due to differential coactivator recruitment and is characterized as selective PPARγ modulator (sPPARγM).

## Introduction

With rising incidence, gout is the most prevalent arthritic disease affecting approximately 3% of the population worldwide. It arises from chronic hyperuricaemia leading to disposal of urate microcrystals in and around joints. These urate crystals together with other factors can then induce a painful inflammatory reaction. The disease was long neglected and poorly studied but the recent decade has improved its understanding and yielded several novel therapeutics. According to current knowledge, gout is strongly associated with metabolic diseases such as type 2 diabetes mellitus and the metabolic syndrome^1^. Treating metabolic dysbalance might, therefore, be a future strategy to cure gout.

Late in 2015, the urate transporter inhibitor lesinurad^2^ was approved for the treatment of gout and expected to gain a dominant role in gout therapy. It acts as uricosuric agent by inhibiting the uric acid re-absorbing solute carrier SLC22A12 also known as URAT1^3^ in the kidney and thereby promotes urinary uric acid excretion^2^. In clinical development, lesinurad was studied in combination therapy with allopurinol^4^ or febuxostat^5^ and as monotherapy^6^ and in all trials markedly decreased serum uric acid levels with a generally favorable safety profile. However, indicators for increased cardiovascular risk were observed at higher lesinurad doses^7^ that may point to off-target activities. Beyond that, clinically more relevant nephrotoxicity has led to a black box warning about acute renal failure under lesinurad therapy^8^.

The peroxisome proliferator-activated receptor γ (PPARγ) acts as fatty acid sensing nuclear receptor and has a long history as drug target^9^. PPARγ is a key regulator of adipocyte differentiation and fatty acid storage but also has insulin-sensitizing and anti-inflammatory effects. PPARγ-agonistic thiazolidinediones (rosiglitazone, pioglitazone) reached blockbuster status as oral antidiabetics until safety warnings concerning increased cardiovascular risk and bladder cancer incidence significantly lowered their therapeutic relevance^10^. In addition, weight gain as a common side-effect of PPARγ agonists is an obstacle for their use especially in metabolic diseases. Still, PPARγ stays in focus of drug discovery for a variety of indications including metabolic^11^ and inflammatory^12^ disorders. Recent progress in PPARγ research has yielded selective PPARγ modulators (reviewed in^11^) that exploit beneficial effects of PPARγ activation *in vivo* while partly avoiding its adverse effects.

Due to its remarkable structural similarity with known PPARγ ligands, we have studied the activity of lesinurad on PPARs and several related nuclear receptors and conclude that the recently approved uricosuric agent exhibits selective PPARγ-modulatory activity that may significantly contribute to its therapeutic efficacy in gout.

## Results

### Molecular Docking

**Figure 1:**
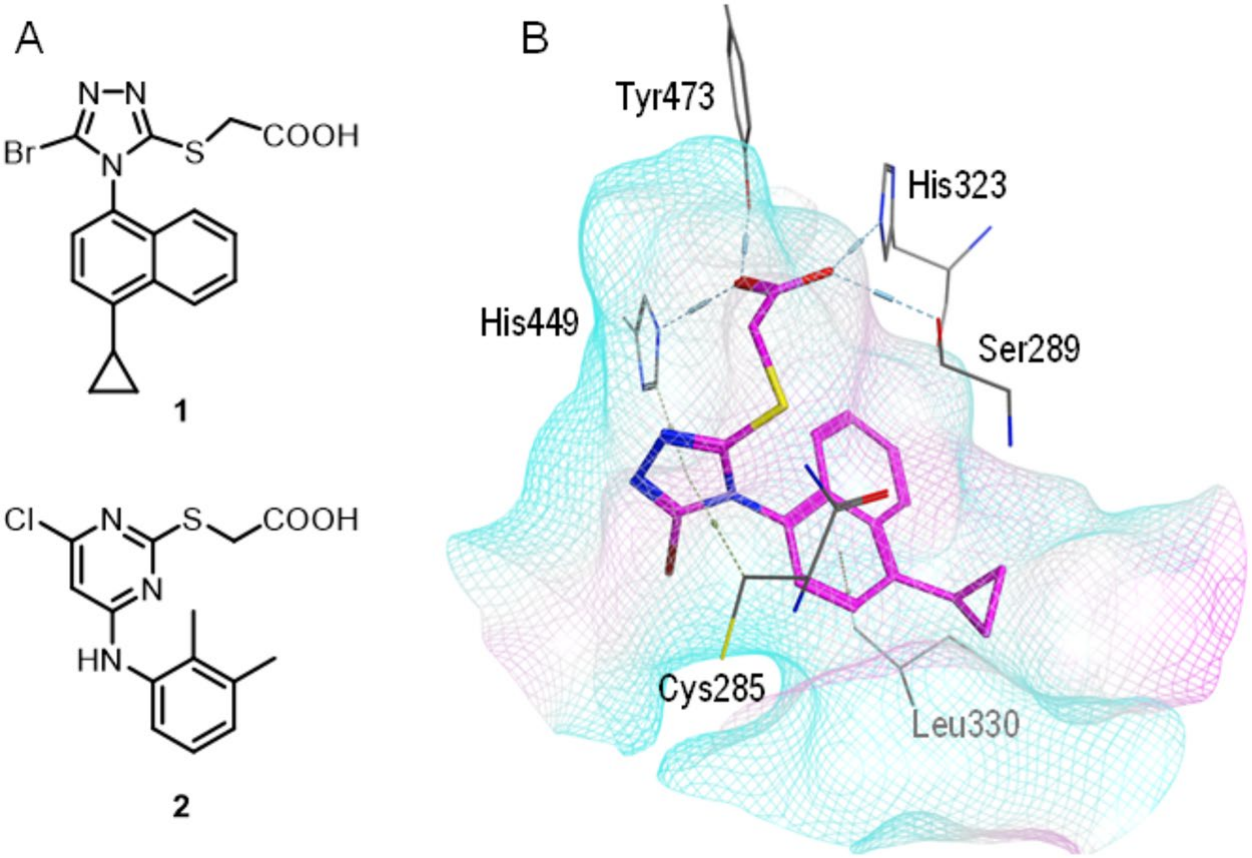
(**A**) Lesinurad (**1**) structurally resembles the dual PPARα/PPARγ agonist WY14,643 (**2**) and shares the typical architecture of fatty acid mimetics. (**B**) Molecular docking of lesinurad (**1**) into the PPARγ ligand binding domain (PDB-ID: 3ET3^17^) suggests a very favorable binding mode. The carboxylic acid of **1** forms neutralizing contacts with the triad of Ser_289_, His_323_, His_449_ and Tyr_473_ and the triazole moiety participates in π-interactions with Cys_285_ and His_449_. Lesinurad’s hydrophobic tail is placed in a lipophilic arm of the PPARγ ligand binding site.

Lesinurad (**1**)^2^ comprises the typical molecular architecture of fatty acid mimetics^13^ and shares considerable structural similarity with the PPAR agonist WY14,643^14–16^ (**2**, Fig. 1A). Molecular docking of lesinurad into the PPARγ ligand binding site (PDB ID: 3ET3^17^, Fig. 1B) suggested a very favorable binding mode involving various ligand-protein interactions. The carboxylic acid of the drug participated in a hydrogen bond network with Ser_289_, His_323_, His_449_ and Tyr_473_, which is common for PPARγ agonists. Moreover, the triazole ring of lesinurad formed π-interactions with Cys_285_ and His_449_.

### Reporter Gene Assay

**Figure 2:**
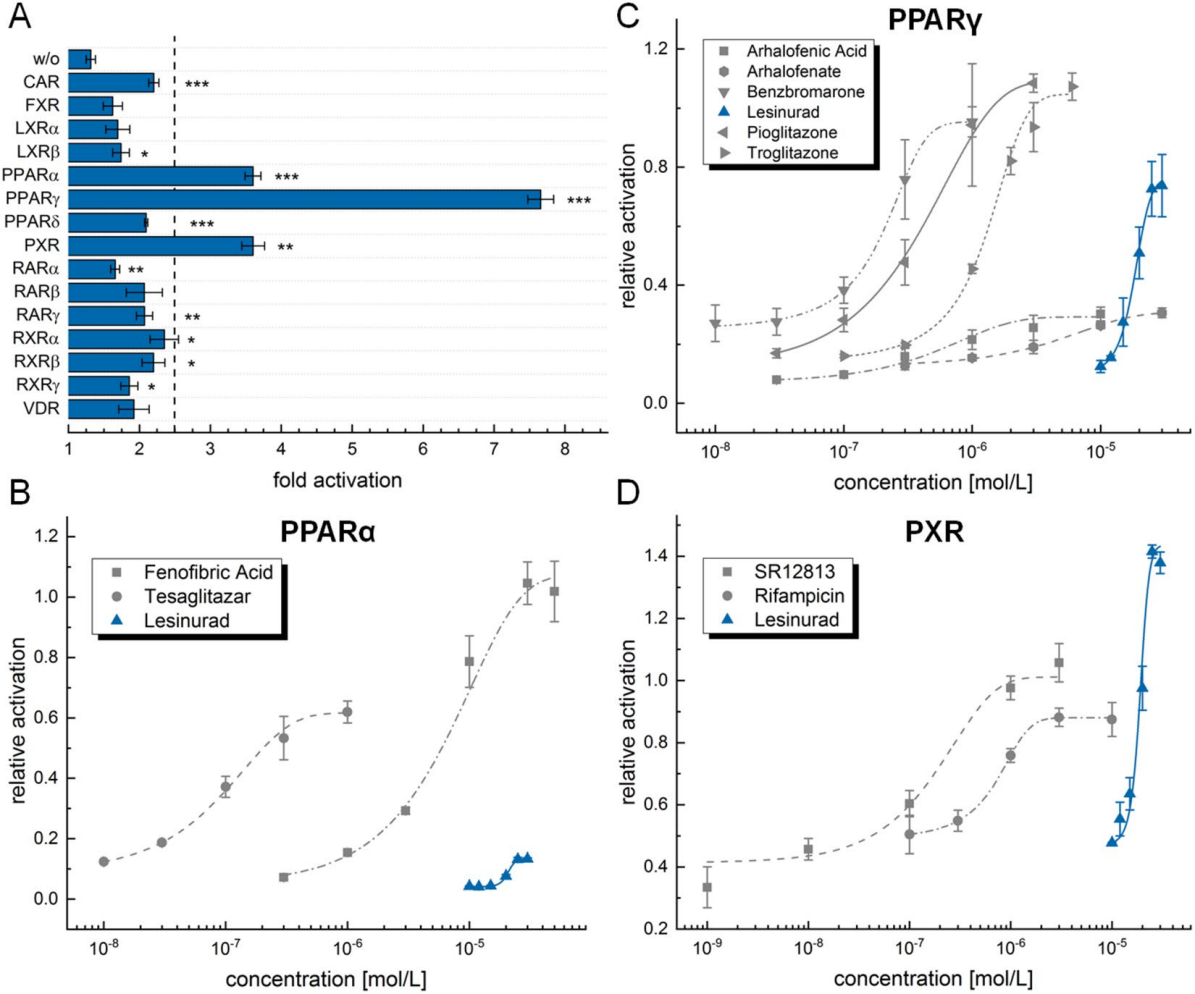
*In vitro* profiling of lesinurad on nuclear receptors: (**A**) At 30 μM concentration, lesinurad robustly transactivated Gal4-PPARγ and - with lower efficacy - Gal4-PPARα and Gal4-PXR. No reporter transactivation was observed in absence (w/o) of a hybrid receptor confirming nuclear receptor-mediated activity. Effects below a fold activation of 2.5 (dashed line) were considered irrelevant despite statistical significance. (**B**–**D**) Dose-response curves of lesinurad (blue) and selected drugs/reference compounds (grey) for comparison on PPARα (**B**), PPARγ (**C**) and PXR (**D**): (**B**) Transactivation efficacy of lesinurad on PPARα is very modest and, thus, this activity appears neglectable. (**C**) Dose-response characterization of lesinurad on PPARγ revealed an EC_50_ value of 18.5 ± 0.7 μM and 79 ± 5% relative efficacy compared to the reference PPARγ agonist pioglitazone. (**D**) On PXR, lesinurad possessed high transactivation efficacy (145 ± 9% compared to SR12813) which is not surprising since it has been characterized as CYP3A4 inducer previously^20^. Results are mean ± SEM of at least three independent experiments; *p < 0.05, **p < 0.01, ***p < 0.001.

Intrigued by this observation, we profiled lesinurad on PPARs and twelve closely related nuclear receptors in specific Gal4 hybrid reporter gene assays (Fig. 2A). These *in vitro* test systems are based on a constitutively expressed hybrid receptor composed of the ligand binding domain of the respective human nuclear receptor and the DNA binding domain of the yeast receptor Gal4. A Gal4-inducible firefly luciferase serves as reporter gene and a constitutively expressed renilla luciferase is used for normalization of transfection efficacy and internal toxicity control^18,19^. In these test systems, lesinurad at 30 μM concentration robustly transactivated PPARγ and, despite with lower efficacy, PPARα and pregnane X receptor (PXR). Control experiments in absence of a hybrid receptor showed no reporter transactivation confirming that the effects of lesinurad were mediated by the human nuclear receptor ligand binding domains. Full dose-response characterization revealed an EC_50_ value of 18.5 ± 0.7 μM for lesinurad on PPARγ combined with almost equal efficacy as the full PPARγ agonist pioglitazone (Fig. 2C). On PPARα, lesinurad possessed a similar EC_50_ value (20.8 ± 0.7 μM) but markedly lower activation efficacy (13.5 ± 0.5% vs. 1 µM of PPARα agonist GW7647). Compared to the remarkable PPARγ-agonistic activity, this weak partial PPARα agonism appears less relevant. In addition, PXR-agonistic activity (EC_50_ = 19 ± 1 μM, 145 ± 9% efficacy vs. 1 µM SR12813) was observed but was not surprising since the drug has been characterized as CYP3A4 inducer previously^20^.

The marked PPARγ-agonistic activity of lesinurad according to reported plasma concentrations of up to 29 µM^21^ (200 mg p.o.) and 54 μM^21^ (600 mg p.o.) might indeed have pharmacological relevance especially since PPARγ activation might beneficially support lesinurad’s mode of action. Therefore, we further characterized this pharmacodynamic effect by various biophysical and *in vitro* pharmacological studies.

First, we compared the activities of lesinurad with several drugs known to modulate PPARα, PPARγ or PXR (Figure 2B–D, Table 1). For PPARα agonist fenofibric acid, PPARγ activators pioglitazone, rosiglitazone and troglitazone as well as PXR agonists SR12813 and rifampicin, our hybrid reporter gene assays clearly reproduced the potencies of these reference drugs in terms of EC_50_ values and transactivation efficacy as reported in literature. For arhalofenic acid and tesaglitazar, we observed discrepancies between reported PPARγ potency^22,23^ and behavior in our test system that is well explained by the fact that the literature activities refer to murine PPARγ. The determined potency of tesaglitazar on PPARα also exceeds the reported value which may be due to marked differences in assay conduct. The literature activity^23^ of tesaglitazar refers to a hybrid transactivation assay that in contrast to our system involves a very long incubation period of 40 hours (vs 12–14 hours in our protocol) which might cause significant degradation of the test compound leading to underestimation of its potency. Overall, potencies and transactivation efficacies of the reference drugs observed in our test system agreed very well with literature which further validated the assay systems and the activity of lesinurad. As further reference, we also compared the activities of lesinurad and urate transporter inhibitor benzbromarone which was characterized as PPARγ activator previously^24^. Benzbromarone revealed similar activation efficacy but significantly higher potency than lesinurad on PPARγ.

**Table 1:** *In vitro* activities of lesinurad and PPAR/PXR activating reference compounds/drugs for comparison.

drug	PPARα (EC_50_ [µM])		PPARγ (EC_50_ [µM])			PXR (EC_50_ [µM])	
	Gal4 assay	literature	Gal4 assay	full-length assay	literature	Gal4 assay	literature
arhalofenate*			5.4 ± 1.1		—		
arhalofenic acid*			0.47 ± 0.02		12^22^**		
benzbromarone			0.21 ± 0.03		active^24^***		
fenofibrate*	inactive	—					
fenofibric acid*	6.5 ± 0.5	30^56^					
pioglitazone			0.41 ± 0.09	1.7 ± 0.1	0.58^56^		
rosiglitazone			0.052 ± 0.010	0.39 ± 0.03	0.043^56^		
tesaglitazar	0.099 ± 0.005	1.7^23^	0.049 ± 0.005		0.25^23^**		
troglitazone			1.5 ± 0.1		0.55^56^		
SR12813						0.17 ± 0.02	0.14^57^
rifampicin						0.76 ± 0.07	0.72^57^
**lesinurad**	**20**.**8 ± 0**.**7**	**—**	**18**.**5 ± 0**.**7**	**21 ± 2**	—	**19 ± 1**	—

Where available, literature values refer to reporter gene assays similar to our test systems. However, there are differences concerning incubation times and used cell lines that may affect observed activities. Results are mean ± SEM of at least three independent experiments.

*Arhalofenate and fenofibrate are prodrugs with arhalofenic acid and fenofibric acid as active metabolites.

**Literature values of arhalofenic acid and tesaglitazar refer to murine PPARγ.

***No precise activity value has been reported for benzbromarone on PPARγ.

### Full-length PPARγ reporter gene assay

**Figure 3:**
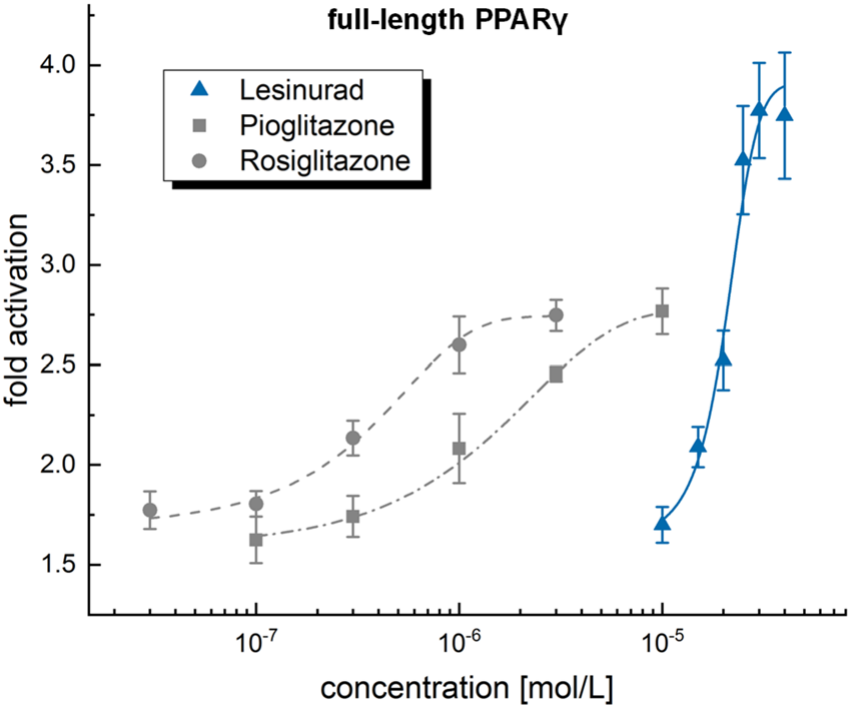
Activity of lesinurad in a full-length PPARγ reporter gene assay compared to reference agonists rosiglitazone and pioglitazone: With an EC_50_ value of 21 ± 2 µM, lesinurad was equally potent on full-length PPARγ as on the hybrid receptor and revealed higher transactivation efficacy than pioglitazone and rosiglitazone. Results are mean ± SEM of at least three independent experiments; *p < 0.05, **p < 0.01, ***p < 0.001.

The activity we observed for lesinurad in the hybrid reporter gene assay was reproduced in a full-length PPARγ transactivation assay involving the entire human nuclear receptor (Fig. 3). In this less artificial test system, lesinurad revealed equal potency with an EC_50_ value of 21 ± 2 µM confirming that lesinurad is competent to activate the human full-length PPARγ protein. The potencies of pioglitazone (EC_50_ = 1.7 ± 0.1 µM) and rosiglitazone (EC_50_ = 0.39 ± 0.03 µM) in this assay agree with their activities reported in literature which validated the test system and the activity observed for lesinurad (Table 1).

### Isothermal titration calorimetry

**Figure 4:**
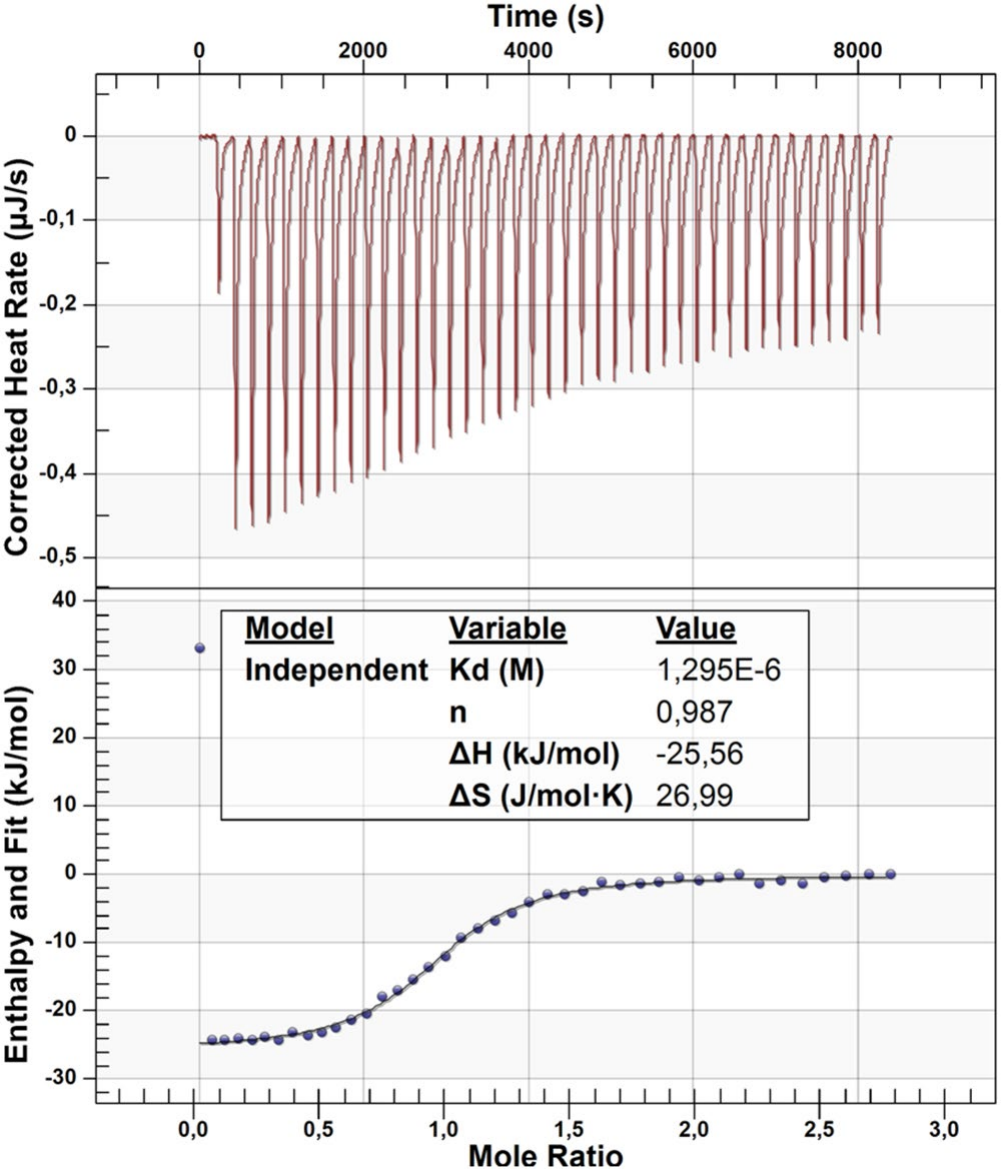
Isothermal titration calorimetry (ITC, recombinant PPARγ ligand binding domain was titrated to lesinurad due to the drug’s limited aqueous solubility) confirmed direct interaction of lesinurad with the PPARγ ligand binding domain and revealed an independent K_d_ value of 1.3 μM. Binding was driven by enthalpic (ΔH = −26 kJ/mol) and entropic (ΔS = 27 J/mol·K) shares confirming the proposed binding mode which revealed two H-bonds and several lipophilic contacts.

To confirm direct modulation of PPARγ by lesinurad, we analyzed their interaction by isothermal titration calorimetry (ITC). Due to limited solubility of lesinurad, recombinant PPARγ ligand binding domain (LBD) protein was (reversely) titrated to the drug. The ITC experiment confirmed direct high-affinity binding of lesinurad to the PPARγ LBD with an independent K_d_ value of 1.3 μM (Fig. 4). Moreover, ITC data indicated that lesinurad binding to PPARγ is driven by both enthalpic (ΔH = −26 kJ/mol) and entropic (ΔS = 27 J/mol·K) contributions. The enthalpic share of −26 kJ/mol equals two hydrogen bonds as suggested by the molecular docking.

### Differentiation of 3T3-L1 adipocytes and PPAR-regulated gene expression

**Figure 5:**
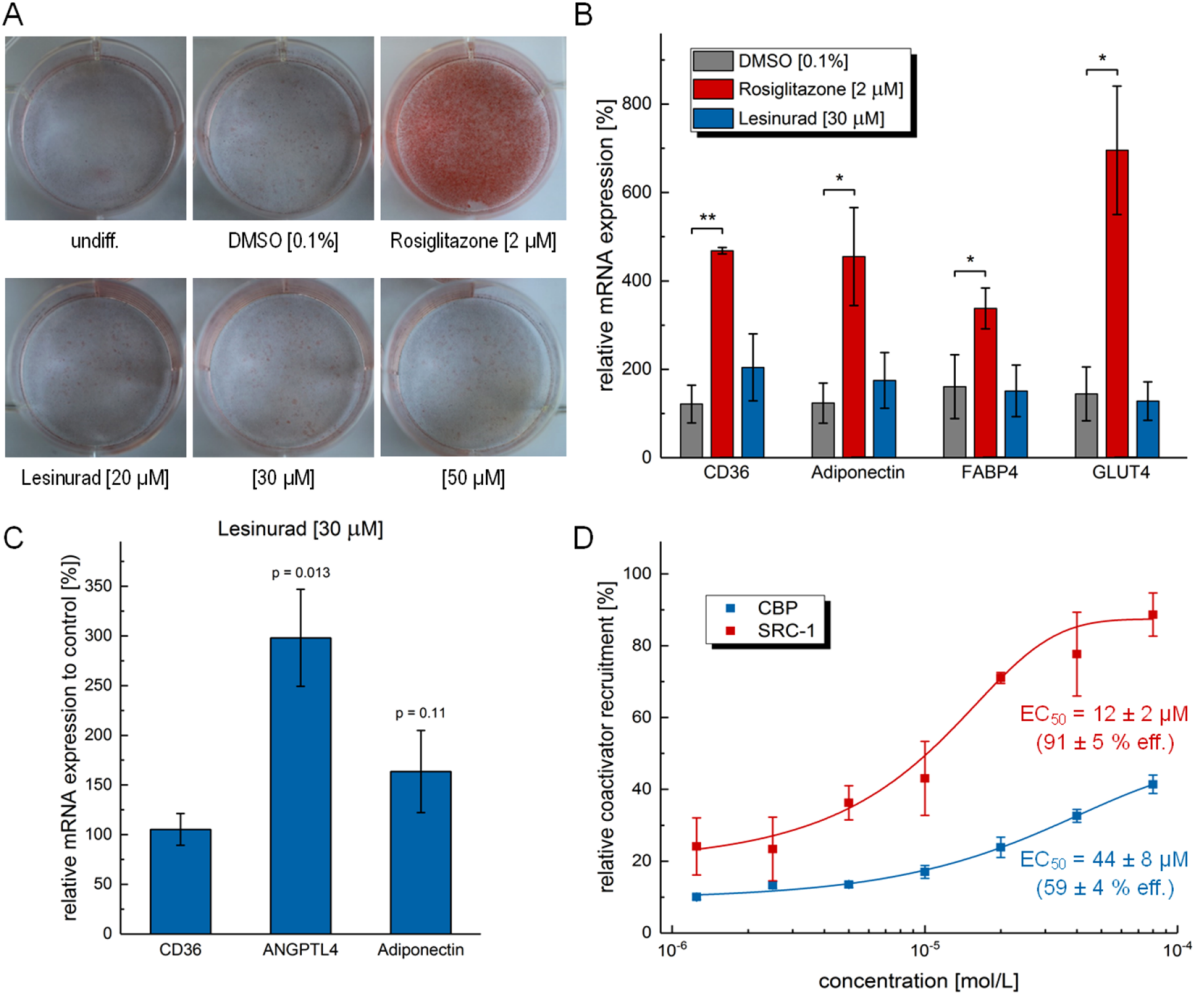
*In vitro* pharmacological characterization of lesinurad: (**A**) Despite its PPARγ-agonistic potency, lesinurad does not induce fat accumulation in murine 3T3-L1 adipocytes up to a concentration of 50 µM whereas the classical PPARγ agonist rosiglitazone causes massive storage of fat visualized by Oil Red O staining. (**B**) Rosiglitazone robustly induces PPARγ-regulated genes scavenger receptor CD36, adiponectin, fatty acid binding protein 4 (FABP4) and glucose transporter 4 (GLUT4) in 3T3-L1 adipocytes whereas lesinurad hardly affects their expression. (**C**) In HepG2 cells, lesinurad did not alter the expression of CD36 and only slightly induced adiponectin but caused marked induction of angiopoietin-like 4 (ANGPTL4). (**D**) Lesinurad robustly recruits the steroid receptor coactivator 1 (SRC-1) with an EC_50_ value of 12 ± 2 μM and 91 ± 5% efficacy compared to 1 μM rosiglitazone. In contrast, recruitment of CREB binding protein (CBP) is significantly weaker induced by lesinurad (EC_50_ = 44 ± 8 μM; 59 ± 4% efficacy compared to 1 μM rosiglitazone). Results are mean ± SEM of at least three independent experiments; *p < 0.05, **p < 0.01, ***p < 0.001.

As PPARγ is a major regulator of lipid homeostasis and adipocyte differentiation, we studied the effects of lesinurad on lipid accumulation in murine 3T3-L1 adipocytes. For this, 3T3-L1 cells were treated with insulin, dexamethasone and isobutylmethylxanthine (IBMX) to induce their differentiation into mature adipocytes. In this experiment, addition of the synthetic PPARγ agonist rosiglitazone at 2 μM caused massive accumulation of lipid droplets during differentiation visualized by Oil Red O staining whereas lesinurad up to a concentration of 50 μM lacked this adipogenic effect. Cells treated with lesinurad were indistinguishable from DMSO-treated cells and even undifferentiated controls (Fig. 5A). Of note, lesinurad exhibited no cytotoxicity up to 50 μM (data not shown) excluding that the lack of lipid accumulation is a toxic effect. To gain deeper understanding of lesinurad-mediated PPARγ modulation in adipocytes, we analyzed their gene expression profiles after differentiation (Fig. 5B). Compared to DMSO-treated cells, the full PPARγ agonist rosiglitazone robustly induced the scavenger receptor CD36, adiponectin, fatty acid binding protein 4 (FABP4) and the glucose transporter 4 (GLUT4). In strong contrast, lesinurad at 30 μM caused almost no changes in PPARγ-regulated gene expression with only slight trends for CD36 and adiponectin induction. Thus, the gene expression profiles confirmed the results of the staining experiments and indicated that lesinurad does not activate pro-adipogenic PPARγ target gene transcription in adipocytes to cause lipid accumulation. In contrast, in human hepatoma cells (HepG2 cells), lesinurad caused a more distinguished effect on PPARγ-regulated genes (Fig. 5C). As in adipocytes, lesinurad hardly affected CD36 and adiponectin expression but markedly induced angiopoietin-like 4 (ANGPTL4).

Thus, the cellular gene expression profiles indicated that lesinurad differentially affects distinct PPARγ-regulated genes which could be due to differential coactivator recruitment.

### Coactivator recruitment assay

We analyzed the ability of lesinurad to induce recruitment of the coactivators steroid receptor coactivator 1 (SRC-1, also known as nuclear receptor coactivator 1 (NCOA1)) and cyclic AMP responsive element binding (CREB) protein (CBP) to the PPARγ ligand binding domain in a time-resolved FRET assay with labeled PPARγ-LBD and coactivator proteins (Fig. 5D). Lesinurad robustly induced the recruitment of SRC-1 with an EC_50_ value of 12 ± 2 μM and 91 ± 5% efficacy compared to rosiglitazone (1 μM). In contrast, lesinurad-induced CBP recruitment was significantly weaker in terms of EC_50_ value (44 ± 8 μM) and relative efficacy (59 ± 4%). According to this, at the concentrations of lesinurad used in our *in vitro* studies and at physiological concentrations, recruitment of CBP plays only a minor role.

## Discussion

Lesinurad has been developed and approved as inhibitor of the urate transporter 1 (URAT1) on which it possesses intermediate potency with an IC_50_ value of 3.5 μM^2^. According to our results, **1** with an EC_50_ value of 18.5 μM has almost equal potency as PPARγ modulator and therefore potentially acts as gout therapeutic by a dual mode of action.

We have characterized the interaction of lesinurad with the PPARγ ligand binding domain employing a broad spectrum of *in vitro* test systems. A hybrid reporter gene assay that was validated with numerous reference compounds revealed strong PPARγ transactivation efficacy of lesinurad and ITC experiments clearly assigned this effect to a direct interaction of the drug with the PPARγ ligand binding domain. Equal potency of lesinurad in a full-length PPARγ transactivation assay confirmed that the drug is also competent to activate the native human nuclear receptor and that the observed activities were not an artifact of the test systems relying on the PPARγ ligand binding domain.

Contradictory to our data, Miner *et al*.^2^ have reported that lesinurad does not activate PPARγ up to 100 μM concentration. This discrepancy might be explained by the fact that Miner *et al*. used a (not further specified) non-human cell line overexpressing PPARγ and engineered with a PPAR-responsive luciferase reporter. This cell line might either express a non-human PPARγ that is not activated by lesinurad or lacks human coactivators that are required for PPARγ activity. In all our test systems except the murine adipocyte cell line 3T3-L1, human cell lines and human PPARγ was used including recombinant human PPARγ ligand binding domain protein for the ITC measurement and coactivator recruitment assay as well as a hybrid receptor containing the human PPARγ ligand binding domain in the transactivation assay. PPARγ is highly conserved between human and mouse and it is commonly agreed that the mouse is a suitable species for PPARγ-related experiments^25^. Thus, we conclude that lesinurad is an intermediately potent agonist of PPARγ with high transactivation efficacy.

Gout is characterized by high concentrations of uric acid leading to disposal of urate crystals that cause an inflammatory arthritis^26^. The disease is associated with metabolic disorders, particularly the metabolic syndrome which makes a relation to PPARγ obvious^1^. Current international guidelines^26–30^ for the management of gout recommend weight-lowering lifestyle changes and the supportive treatment of metabolic dysbalance and cardiovascular risk factors such as type 2 diabetes mellitus, dyslipidemia and obesity. In human HepG2 cells, lesinurad markedly induced expression of angiopoetin-like protein 4 (ANGPTL4) which is involved in glucose homeostasis, lipid metabolism and might improve insulin sensitivity^31,32^. Moreover, lesinurad had a tendency to induce adiponectin that functions as a regulator of glucose levels and fatty acid degradation^33^. Gout patients with metabolic comorbidities such as type 2 diabetes mellitus and other manifestations of the metabolic syndrome may therefore strongly profit from PPARγ activation also in terms of reduced gout. In addition, PPARγ is a major regulator of fatty acid uptake and storage which strongly contributes to its beneficial metabolic effects^34–36^. Recent progress in understanding gout pathology points to an important role of fatty acids as cofactor of urate microcrystals in causing inflammation^1^. Improved fatty acid homeostasis upon PPARγ activation might have beneficial impact in gout treatment. Moreover, as inflammatory disease, gout may also benefit from other anti-inflammatory effects of PPARγ activation. The PPARγ agonist pioglitazone is studied in clinical trials^12,37,38^ for rheumatoid arthritis (as well as other chronic inflammatory diseases) where it shows promising activity especially in combination therapy with standard of care. In addition, direct effects of pioglitazone in a rat model of gout have been reported and associated with reduced synovial cytokine (TNFα, IFN-γ and IL-1β) levels^39^. Therefore, the PPARγ-agonistic potency of lesinurad seems to support its URAT1-inhibitory activity which together form a convenient dual mode of action: while URAT1 inhibition controls uric acid levels and prevents acute inflammatory episodes, PPARγ activation is a validated and efficacious therapeutic approach to metabolic diseases and in addition may particularly reduce the chronic inflammatory aspect of gout.

Pharmacokinetic studies^21^ of lesinurad in healthy men reported mean peak plasma levels of 22 μg/mL after a single 600 mg oral dose corresponding to 54 μM plasma concentration. For the commonly used oral dose of 200 mg reported peak plasma levels vary between 15 µM^40^, 17 µM^41^ and 29 μM^21^ plasma concentration. High (98%) plasma protein binding^2,41^ has been observed for lesinurad and this interaction would lower the free concentration to approximately 0.3–0.6 µM^2^, normally precluding a clinical effect. However, lesinurad is known to be a substrate of various organic solute transporters^41^ and as other fatty acid mimetics^13^ thus could accumulate in cells providing the potential for PPARγ modulation in humans.

Classical PPARγ agonists such as the thiazolidinediones rosiglitazone and pioglitazone are commonly characterized by adipogenic effects translating into weight gain as clinical side-effect. Extensive research has focused on separating this effect from the variety of beneficial activities mediated by the nuclear receptor culminating in the development of selective PPARγ modulators (sPPARγM). Of note, the term “selective” here refers to the mode of PPARγ modulation in terms of tissue-selectivity or selective effects compared to full PPARγ agonists such as pioglitazone but not to the selectivity over related molecular targets such as other PPAR subtypes. Several sPPARγMs^11,42,43^ have been developed that differentially induce PPARγ-regulated genes potentially due to differential recruitment of different coactivators to the nuclear receptor. According to our results, lesinurad also constitutes such sPPARγM and is free from the adipogenic effect of classical PPARγ agonists *in vitro*. This profile further supports lesinurad’s PPARγ-modulatory activity as beneficial contributor to the drug’s pharmacological profile.

In murine adipose tissue, expression of both SRC-1 and CBP was observed^44^ but CBP/PPARγ interaction was shown crucial for differentiation of 3T3-L1 adipocytes^45,46^ which agrees with our observation that lesinurad does neither induce CBP recruitment nor promote adipocyte differentiation or lipid accumulation in 3T3-L1 cells. In contrast, SRC-1 which is robustly recruited to PPARγ through lesinurad binding has been associated with the regulation of energy homeostasis in adipose tissue and SRC-1 knockout in mice favored obesity development^47^. Additionally, SRC-1 plays a major role in thermogenesis^47^. Together, this data suggests that a PPARγ ligand that recruits SRC-1 but not CBP to the PPARγ ligand binding domain causes beneficial metabolic effects without inducing adipogenesis.

The selective modulatory activity of lesinurad on PPARγ might, therefore, also catch attention for selective optimization of side-activities. As approved drug, lesinurad is safe and bioavailable and therefore a very valuable starting point for new drug development. Minor structural variations might allow optimizing the selective PPARγ-modulatory characteristic and reduce URAT1 inhibition to generate a more potent sPPARγM for novel indications in metabolic diseases.

The predecessor of lesinurad and structurally related uricosuric benzbromarone has been characterized as PPARγ agonist previously^24^. This activity was confirmed in our hybrid reporter gene assay where we observed significantly higher PPARγ-agonistic potency for benzbromarone (EC_50_ = 0.21 µM) compared to lesinurad (EC_50_ = 18.5 µM) but equal transactivation efficacy. As a result, benzbromarone enhanced adiponectin plasma levels in men^48^, but, in contrast to lesinurad, benzbromarone also significantly induced the expression of CD36, adiponectin and FABP4 (aP2) in 3T3-L1 mouse fibroblasts^48^ where lesinurad showed no effect in our study. Benzbromarone, therefore, appears to promote adipocyte differentiation and to have adipogenic potential. In HEK293T cells, benzbromarone induced lipoprotein lipase (LPL) and acyl-CoA-synthetase^24^ suggesting PPAR activation in this cell type, as well. Together these results indicate marked differences in the mode of PPARγ modulation by lesinurad and benzbromarone and suggest that lesinurad but not benzbromarone has selective PPARγ-modulatory properties. Still, the PPARγ-agonistic activity of both uricosuric drugs may contribute to therapeutic efficacy in gout in a dual mode of action. The lack of adipogenic effects appears as a potential safety advantage of lesinurad, however.

In conclusion, we report a secondary mode of action for the recently approved gout therapeutic lesinurad. By selective PPARγ modulation and differential induction coactivator recruitment to the PPARγ ligand binding domain, lesinurad activates PPARγ without causing adipogenic effects *in vitro*. As PPARγ activation beneficially affects chronic inflammatory diseases such as rheumatoid arthritis, and as gout is closely associated with metabolic diseases, lesinurad’s PPARγ modulation appears as supportive side-activity to its URAT1 inhibitory potency. Moreover, with its interesting pharmacological profile, lesinurad might also hold great therapeutic potential in metabolic diseases since adipogenic effects of PPARγ activation and resulting weight gain are a major obstacle to the therapeutic exploitation of PPARγ in metabolic diseases. By avoiding this adipogenic activity, lesinurad could help PPARγ to a rebirth as target for metabolic disorders or serve as lead compound for the development of potent drug-like sPPARγM by selective optimization of side-activities^49^.

## Materials and Methods

### Hybrid reporter gene assays for PPARα/γ/δ, LXRα/β, RXRα/β/γ, RARα/β/γ, FXR, VDR, CAR and PXR

#### Plasmids

The Gal4-fusion receptor plasmids pFA-CMV-hPPARα-LBD^50^, pFA-CMV-hPPARγ-LBD^50^, pFA-CMV-hPPARδ-LBD^50^, pFA-CMV-hLXRα-LBD^51^, pFA-CMV-hLXRβ-LBD^51^, pFA-CMV-hRXRα-LBD^52^, pFA-CMV-hRXRβ-LBD^52^, pFA-CMV-hRXRγ-LBD^52^, pFA-CMV-hRARα-LBD^52^, pFA-CMV-hRARβ-LBD^52^, pFA-CMV-hRARγ-LBD^52^, pFA-CMV-hFXR-LBD^53^, pFA-CMV-hVDR-LBD^52^, pFA-CMV-hCAR-LBD^52^ and pFA-CMV-hPXR-LBD^52^ coding for the hinge region and ligand binding domain (LBD) of the canonical isoform of the respective nuclear receptor have been reported previously. pFR-Luc (Stratagene) was used as reporter plasmid and pRL-SV40 (Promega) for normalization of transfection efficiency and cell growth.

#### Assay procedure

HEK293T cells were grown in DMEM high glucose, supplemented with 10% FCS, sodium pyruvate (1 mM), penicillin (100 U/mL) and streptomycin (100 μg/mL) at 37 °C and 5% CO_2_. The day before transfection, HEK293T cells were seeded in 96-well plates (3·10^4^ cells/well). Before transfection, medium was changed to Opti-MEM without supplements. Transient transfection was carried out using Lipofectamine LTX reagent (Invitrogen) according to the manufacturer’s protocol with pFR-Luc (Stratagene), pRL-SV40 (Promega) and the corresponding Gal4-fusion nuclear receptor plasmid. 5 h after transfection, medium was changed to Opti-MEM supplemented with penicillin (100 U/mL), streptomycin (100 μg/mL), now additionally containing 0.1% DMSO and the respective test compound or 0.1% DMSO alone as untreated control. Each concentration was tested in duplicates and each experiment was repeated independently at least three times. Following overnight (12-14 h) incubation with the test compounds, cells were assayed for luciferase activity using Dual-Glo™ Luciferase Assay System (Promega) according to the manufacturer’s protocol. Luminescence was measured with an Infinite M200 luminometer (Tecan Deutschland GmbH). Normalization of transfection efficiency and cell growth was done by division of firefly luciferase data by renilla luciferase data and multiplying the value by 1000 resulting in relative light units (RLU). Fold activation was obtained by dividing the mean RLU of a test compound at a respective concentration by the mean RLU of untreated control. Relative activation was obtained by dividing the fold activation of a test compound at a respective concentration by the fold activation of a respective reference agonist at 1 μM (PPARα: GW7647; PPARγ: pioglitazone; PPARδ: L165,041; LXRα/β: T0901317; RXRα/β/γ: bexarotene; RARα/β/γ: tretinoin; FXR: GW4064; VDR: calcitriol; CAR: CITCO; PXR: SR12813). All hybrid assays were validated with the above-mentioned reference agonists which yielded EC_50_ values in agreement with literature.

### Full-length PPARγ reporter gene assay

HEK293T cells were grown in DMEM high glucose, supplemented with 10% FCS, sodium pyruvate (1 mM), penicillin (100 U/mL) and streptomycin (100 μg/mL) at 37 °C and 5% CO_2_. The day before transfection, HEK293T cells were seeded in 96-well plates (3·10^4^ cells/well). Before transfection, medium was changed to Opti-MEM without supplements. Transient transfection was carried out using Lipofectamine LTX reagent (Invitrogen) according to the manufacturer’s protocol with hPPARγ responsive firefly luciferase construct PPRE1-pGL3 and pRL-SV40 (Promega) as internal control to monitor transfection efficiency and test compound toxicity. 5 h after transfection, medium was changed to Opti-MEM supplemented with penicillin (100 U/mL), streptomycin (100 μg/mL), now additionally containing 0.1% DMSO and the respective test compound or 0.1% DMSO alone as untreated control. Each concentration was tested in duplicates and each experiment was repeated independently at least three times. Following overnight (12-14 h) incubation with the test compounds, cells were assayed for luciferase activity using Dual-Glo™ Luciferase Assay System (Promega) according to the manufacturer’s protocol. Luminescence was measured with an Infinite M200 luminometer (Tecan Deutschland GmbH). Normalization of transfection efficiency and cell growth was done by division of firefly luciferase data by renilla luciferase data and multiplying the value by 1000 resulting in relative light units (RLU). Fold activation was obtained by dividing the mean RLU of a test compound at a respective concentration by the mean RLU of untreated control. The assay was validated with reference agonists pioglitazone and rosiglitazone which yielded EC_50_ values in agreement with literature.

### Isothermal titration calorimetry (ITC)

Isothermal titration calorimetry (ITC) was conducted on an Affinity ITC (TA Instruments Affinity ITC). Recombinant PPARγ ligand binding domain protein (138 μM) and lesinurad (40 μM) were each dissolved in a HEPES buffer (25 mM; adjusted to pH 7.5 with KOH; further containing 150 mM KF, 10% glycerol (w/w), 1% DMSO (v/v) and 5 mM DTT). The ITC instrument was adjusted to a temperature of 25 °C and the stirring rate was set at 75 rpm. 190 μL lesinurad solution were filled into the reaction cell and PPARγ ligand binding domain solution was titrated in 41 injections. The first injection had a reduced volume of 1.0 μL and was followed by 40 injections of 2.5 μL. An interval of 200 s was maintained between individual injections. The heats of dilution resulting from titrating PPARγ ligand binding domain protein solution into the buffer solution were recorded in an additional ITC run and subtracted from the raw ITC data obtained for lesinurad.

ITC raw data was analyzed using NanoAnalyze software package (version 3.7.5). An independent binding model was used to fit the reaction enthalpy (Δ*H*), binding affinity constant (*K*_*d*_), and stoichiometry (*n*). Free energy change (Δ*G*) was calculated from the equation Δ*G* = −*RT* ln *K* and the entropy (Δ*S*) was calculated from Δ*G* = Δ*H*−*T*Δ*S*.

### Differentiation of murine 3T3-L1 Cells

3T3-L1 cells were subcultured in DMEM containing 10% newborn calf serum in a humidified atmosphere at 37 °C, 5% CO_2_. Cells were differentiated into adipocytes within 14 days according to the method^54^ reported by Zebisch and colleagues. In brief, cells were seeded in 6-well plates (2.5·10^6^/well). Differentiation was induced at day 3 by addition of 1 μg/mL insulin, 0.25 μM dexamethasone, and 0.5 mM isobutylmethylxanthine as well as the test compounds or controls in DMEM supplemented with 10% fetal calf serum. At day 5, medium was changed to contain only insulin for another two days. Cells were then kept for lipid droplet accumulation in basal medium without additions until day 15. Rosiglitazone (2 μM) was used as PPARγ positive control. Differentiation of 3T3-L1 cells to adipocytes was confirmed by Oil Red O staining. For this, cells were washed with PBS and then fixed for 60 min with a formaldehyde solution (4% in PBS). Subsequently, cells were rinsed with 60% isopropanol and incubated with Oil Red O solution (0.3%) for 120 min. Figure 5A displays one representative staining example of three independent repeats.

### PPARγ target gene quantification

#### 3T3-L1 Adipocytes

3T3-L1 cells were cultured and differentiated as described above. Cells were then lysed using TRIzol reagent (Ambion, Life Technologies, Carlsbad, CA, USA), and mRNA was isolated following the manufacturer’s protocol. DNA contaminations were digested using DNase (DNase I, RNase-free kit; Thermo Scientific, Waltham, MA, USA) and mRNA concentrations were measured with a NanoDrop2000 spectrophotometer (Thermo Scientific, Waltham, MA, USA). Subsequently, reverse transcription was performed using the high capacity RNA-to-cDNA kit (Applied Biosystems, Foster City, CA, USA). Quantitative real-time PCR was performed using specific primers for CD36, Cytokeratin20 and CPT1A with a StepOnePlus real-time PCR system (Applied Biosystems, Foster City, CA, USA). Non-POU domain containing octamer binding protein (NonO) was used as reference gene for 3T3-L1. All samples were measured in duplicate and were analyzed using the ΔΔC_t_ method. The following PCR primers were used: Adiponectin: 5′-TGA CGA CAC CAA AAG GGC TC-3′ (fwd) and 5′-CAC AAG TTC CCT TGG GTG GA-3′ (rev); CD36: 5′-TGC TGG AGC TGT TAT TGG TG-3′ (fwd) and 5′-CAT GAG AAT GCC TCC AAA CA-3′ (rev); FABP4: 5′-AGA AGT GGG AGT GGG CTT TG-3′ (fwd) and 5′-ACT CTC TGA CCG GAT GGT GA-3′ (rev); GLUT4: 5′-TGA AGA ACG GAT AGG GAG CAG-3′ (fwd) and 5′-GAA GTG CAA AGG GTG AGT GAG-3′ (rev); NonO: 5′-TGC TCC TGT GCC ACC TGG TAC TC-3′ (fwd) and 5′-CCG GAG CTG GAC GGT TGA ATG C-3′ (rev).

Results (expressed as mean ± SEM % relative mRNA expression; n = 3): Adiponectin: untreated (0.1% DMSO): 124 ± 45%; rosiglitazone (2 μM): 455 ± 111%; **1** (30 μM): 175 ± 63%; CD36: untreated (0.1% DMSO): 122 ± 43%; rosiglitazone (2 μM): 468 ± 8%; **1** (30 μM): 205 ± 76%; FABP4: untreated (0.1% DMSO): 161 ± 72%; rosiglitazone (2 μM): 338 ± 46%; **1** (30 μM): 151 ± 58%; GLUT4: untreated (0.1% DMSO): 145 ± 61%; rosiglitazone (2 μM): 695 ± 145%; **1** (30 μM): 128 ± 43%.

#### HepG2 cells

HepG2 cells were incubated with **1** (30 μM) or 0.1% DMSO as untreated control for 8 h, harvested, washed with cold phosphate-buffered saline (PBS) and then directly used for RNA extraction. 2 μg total RNA were extracted from HepG2 cells by the Total RNA Mini Kit (R6834-02, Omega Bio-Tek, Inc., Norcross, GA, USA). RNA was reverse-transcribed into cDNA using the High-Capacity cDNA Reverse Transcription Kit (4368814, Thermo Fischer Scientific, Inc.) according to the manufacturer’s protocol. PPARγ target gene expression was evaluated by quantitative real time PCR analysis with a StepOnePlus™ System (Life Technologies, Carlsbad, CA, USA) using Power SYBR Green (Life Technologies; 12.5 μL per well). Each sample was set up in duplicates and repeated in four independent experiments. The expression was quantified by the comparative ∆∆C_t_ method and glycerinaldehyde 3-phosphate dehydrogenase (GAPDH) served as reference gene. The following PCR primers were used: Adiponectin: 5′-TGG CTA TGC TCA CAG TCT CAC ATC-3′ (fwd) and 5′-CTC TGT GCC TCT GGT TCC ACA A-3′ (rev); ANGPTL4: 5′-ATT CTT TCC AGC GGC TTC TG-3′ (fwd) and 5′-GAG GAC TGG AGA CGC GGA G-3′ (rev); CD36: 5′-GGC TGT GAC CGG AAC TGT G-3′ (fwd) and 5′-AGG TCT CCA ACT GGC ATT AGA A-3′ (rev); GAPDH: 5′-ATA TGA TTC CAC CCA TGG CA-3′ (fwd) and 5′-GAT GAT GAC CCT TTT GGC TC-3′ (rev).

Results (expressed as mean ± SEM % relative mRNA expression to vehicle (0.1% DMSO); n = 4): Adiponectin: **1** (30 μM): 163 ± 41%; ANGPTL4: **1** (30 μM): 298 ± 49%; CD36: **1** (30 μM): 105 ± 16%.

### PPARγ coactivator recruitment assay (time-resolved FRET assay)

Recruitment of coactivator-derived peptides to the PPARγ LBD was studied by homogeneous time-resolved fluorescence resonance energy transfer (HT-FRET or HTRF). Terbium cryptate as streptavidin conjugate (cisbio assays, France) was used as FRET donor. Peptides derived from coactivators SRC-1 [biotin-CPSSHSSLTERHKILHRLLQEGSPS] or CBP [biotin-NLVPDAASKHKQLSELLRGGSGS] encompassing the coactivator consensus motif LxxLL and N-terminal biotin for stable coupling to streptavidin were purchased (Eurogentec GmbH, Germany). Solutions containing 12 nM recombinant PPARγ LBD ligand binding domain fused to N-terminal GFP as FRET acceptor and 12 nM of FRET donor complex with either SRC-1 or CBP derived peptide as well as 1% DMSO with test compound at varying concentrations or DMSO alone were prepared in HEPES buffer (25 mM HEPES pH 7.5 adjusted with KOH, 150 mM KF, 5% (w/v) glycerol, 0.1% (w/v) CHAPS and 5 mM DTT). After 2 h incubation at RT, the fluorescence intensities (FI) at 520 nm (acceptor) and 620 nm (donor reference) after excitation at 340 nm were recorded on a Tecan Infinite F200 (Tecan Deutschland GmbH). FI_520 nm_ was divided by FI_620 nm_ and multiplied with 10,000 giving a dimensionless HTRF signal. Coactivator recruitment by the PPARγ LBD brings FRET donor and acceptor into close proximity resulting in a gain in FRET signal as signal for binding. Recruitment of coactivator derived peptides to the PPARγ LBD was validated with increasing concentrations of rosiglitazone (data not shown). Recruitment of SRC-1 or CBP was referenced to recruitment in response to 1 μM rosiglitazone (~EC_80_) and reported as relative coactivator recruitment.

### Molecular docking

Molecular modelling experiments were carried out using MOE (Molecular Operating Environment v. 2016.0802, Chemical Computing Group, Montreal, Canada). Structure preparation of PPARγ LBD (3ET3^17^) was subjected to the Quick Preparation routine, which includes automated structure curation, determination of protonation state and restrained energy minimization. Afterwards, molecular docking was performed using default settings for induced fit docking. London dG scoring function was used for initial placement of 30 poses and afterwards refinement was performed using MM/GBVI method^55^. After visual inspection, the highest-scored pose was selected as basis for the optimization hypothesis.

## References

[1] Pascart, T. & Lioté, F. Gout: state of the art after a decade of developments. *Rheumatology (Oxford)*, 10.1093/rheumatology/key002 (2018). 10.1093/rheumatology/key002 29547895

[2] Miner JN (2016). Lesinurad, a novel, oral compound for gout, acts to decrease serum uric acid through inhibition of urate transporters in the kidney. Arthritis Res. Ther..

[3] Koepsell H, Endou H (2004). The SLC22 drug transporter family. Pflugers Archiv European Journal of Physiology.

[4] Bardin T (2017). Lesinurad in combination with allopurinol: a randomised, double-blind, placebo-controlled study in patients with gout with inadequate response to standard of care (the multinational CLEAR 2 study). Ann. Rheum. Dis..

[5] Dalbeth N (2017). Lesinurad, a Selective Uric Acid Reabsorption Inhibitor, in Combination With Febuxostat in Patients With Tophaceous Gout: Findings of a Phase III Clinical Trial. Arthritis Rheumatol..

[6] Tausche A-K (2017). Lesinurad monotherapy in gout patients intolerant to a xanthine oxidase inhibitor: a 6 month phase 3 clinical trial and extension study. Rheumatology (Oxford)..

[7] Sanchez-Niño MD (2017). Lesinurad: what the nephrologist should know. Clin. Kidney J..

[8] FDA. Zurampic Label. Available at: https://www.accessdata.fda.gov/drugsatfda_docs/label/2017/209203s000lbl.pdf. (Accessed: 21st June 2018).

[9] Lamers C, Schubert-Zsilavecz M, Merk D (2012). Therapeutic modulators of peroxisome proliferator-activated receptors (PPAR): a patent review (2008–present). Expert Opin. Ther. Pat..

[10] Nanjan MJ, Mohammed M, Prashantha Kumar BR, Chandrasekar MJN (2018). Thiazolidinediones as antidiabetic agents: A critical review. Bioorg. Chem..

[11] Gellrich L, Merk D (2017). Therapeutic Potential of Peroxisome Proliferator-Activated Receptor Modulation in Non-Alcoholic Fatty Liver Disease and Non-Alcoholic Steatohepatitis. Nucl. Recept. Res..

[12] Hanke T, Merk D, Steinhilber D, Geisslinger G, Schubert-Zsilavecz M (2015). Small molecules with anti-inflammatory properties in clinical development. Pharmacol. Ther..

[13] Proschak E, Heitel P, Kalinowsky L, Merk D (2017). Opportunities and challenges for fatty acid mimetics in drug discovery. J. Med. Chem..

[14] Santilli AA, Scotese AC, Tomarelli RM (1974). A potent antihypercholesterolemic agent: [4-chloro-6-(2,3-xylidino)-2-pyrimidinylthio]acetic acid (Wy-14643). Experientia.

[15] Pollinger J, Merk D (2017). Therapeutic applications of the versatile fatty acid mimetic WY14643. Expert Opin. Ther. Pat..

[16] Merk D, Zettl M, Steinhilber D, Werz O, Schubert-Zsilavecz M (2015). Pirinixic acids: flexible fatty acid mimetics with various biological activities. Future Med. Chem..

[17] Artis DR (2009). Scaffold-based discovery of indeglitazar, a PPAR pan-active anti-diabetic agent. Proc. Natl. Acad. Sci. USA.

[18] Schmidt J (2017). A dual modulator of farnesoid X receptor and soluble epoxide hydrolase to counter nonalcoholic steatohepatitis. J. Med. Chem..

[19] Merk D, Steinhilber D, Schubert-Zsilavecz M (2014). Characterizing ligands for farnesoid Xreceptor-available *in vitro* test systems for farnesoid Xreceptor modulator development. Expert Opin. Drug Discov..

[20] Gillen M (2017). Evaluation of Pharmacokinetic Interactions Between Lesinurad, a New Selective Urate Reabsorption Inhibitor, and CYP Enzyme Substrates Sildenafil, Amlodipine, Tolbutamide, and Repaglinide. Clin. Pharmacol. Drug Dev..

[21] Shen Z (2015). Pharmacokinetics, pharmacodynamics, and safety of lesinurad, a selective uric acid reabsorption inhibitor, in healthy adult males. Drug Des. Devel. Ther..

[22] Gregoire, F. M. *et al*. MBX-102/JNJ39659100, a novel non-TZD selective partial PPAR-γ agonist lowers triglyceride independently of PPAR-α activation. *PPAR Res*. **2009**, (2009). 10.1155/2009/706852 PMC2673481 19404482

[23] Ljung B (2002). AZ 242, a novel PPARα/γ agonist with beneficial effects on insulin resistance and carbohydrate and lipid metabolism in ob/ob mice and obese Zucker rats. J. Lipid Res..

[24] Kunishima, C. *et al*. Activating effect of benzbromarone, a uricosuric drug, on peroxisome proliferator-activated receptors. *PPAR Res*. **2007** (2007). 10.1155/2007/36092 PMC2233808 18274627

[25] Pap A, Cuaranta-Monroy I, Peloquin M, Nagy L (2016). Is the Mouse a Good Model of Human PPARγ-Related Metabolic Diseases?. Int. J. Mol. Sci..

[26] Jones G, Panova E, Day R (2017). Guideline development for the management of gout: role of combination therapy with a focus on lesinurad. Drug Des. Devel. Ther..

[27] Hui M (2017). The British Society for Rheumatology Guideline for the Management of Gout. Rheumatology.

[28] Richette P (2017). 2016 updated EULAR evidence-based recommendations for the management of gout. Ann. Rheum. Dis..

[29] Dalbeth N (2017). Discordant American College of Physicians and international rheumatology guidelines for gout management: consensus statement of the Gout, Hyperuricemia and Crystal-Associated Disease Network (G-CAN). Nat. Rev. Rheumatol..

[30] Yu, K.-H. *et al*. Management of gout and hyperuricemia: Multidisciplinary consensus in Taiwan. *Int*. *J*. *Rheum*. *Dis*., 10.1111/1756-185X.13266 (2018). 10.1111/1756-185X.13266 29363262

[31] Wang, Y. *et al*. *Molecular medicine reports*. *Molecular Medicine Reports* **14**, (D.A. Spandidos, 2016).

[32] Köster A (2005). Transgenic Angiopoietin-Like (Angptl)4 Overexpression and Targeted Disruption of Angptl4 and Angptl3: Regulation of Triglyceride Metabolism. Endocrinology.

[33] Kern PA, Gregorio GBD, Lu T, Rassouli N, Ranganathan G (2003). Adiponectin Expression From Human Adipose Tissue. Diabetes.

[34] Kersten S, Desvergne B, Wahli W (2000). Roles of PPARs in health and disease. Nature.

[35] Han L, Shen W-J, Bittner S, Kraemer FB, Azhar S (2017). PPARs: regulators of metabolism and as therapeutic targets in cardiovascular disease. Part II: PPAR-β/δ and PPAR-γ. Future Cardiol..

[36] Heikkinen S, Auwerx J, Argmann CA (2007). PPARγ in human and mouse physiology. Biochim. Biophys. Acta - Mol. Cell Biol. Lipids.

[37] Ormseth MJ (2013). Peroxisome proliferator-activated receptor γ agonist effect on rheumatoid arthritis: a randomized controlled trial. Arthritis Res. Ther..

[38] Marder W (2013). The Peroxisome Proliferator Activated Receptor- Pioglitazone Improves Vascular Function and Decreases Disease Activity in Patients With RheumatoidArthritis. J. Am. Heart Assoc..

[39] Wang R-C, Jiang D-M (2014). PPAR-γ agonist pioglitazone affects rat gouty arthritis by regulating cytokines. Genet. Mol. Res..

[40] Astra Zeneca. Zurampic Product Information. Available at: https://www.tga.gov.au/sites/default/files/auspar-lesinurad-160920-pi.docx. (Accessed: 24th June 2018).

[41] Shen Z (2016). *In Vitro* and *In Vivo* Interaction Studies Between Lesinurad, a Selective Urate Reabsorption Inhibitor, and Major Liver or Kidney Transporters. Clin. Drug Investig..

[42] Sohn K-AK (2009). S26948, a new specific peroxisome proliferator activated receptor gamma modulator improved *in vivo* hepatic insulin sensitivity in 48 h lipid infused rats. Eur. J. Pharmacol..

[43] Fujimura T (2005). FK614, a novel peroxisome proliferator-activated receptor gamma modulator, induces differential transactivation through a unique ligand-specific interaction with transcriptional coactivators. J. Pharmacol. Sci..

[44] Yu S, Reddy JK (2007). Transcription coactivators for peroxisome proliferator-activated receptors. Biochimica et Biophysica Acta - Molecular and Cell Biology of Lipids.

[45] Mizukami J, Taniguchi T (1997). The antidiabetic agent thiazolidinedione stimulates the interaction between PPAR gamma and CBP. Biochem. Biophys. Res. Commun..

[46] Takahashi N (2002). Overexpression and Ribozyme-mediated Targeting of Transcriptional Coactivators CREB-binding Protein and p300 Revealed Their Indispensable Roles in Adipocyte Differentiation through the Regulation of Peroxisome Proliferator-activated Receptor. J. Biol. Chem..

[47] Picard F (2002). SRC-1 and TIF2 control energy balance between white and brown adipose tissues. Cell.

[48] Inokuchi T (2009). Effects of benzbromarone and allopurinol on adiponectin *in vivo* and *in vitro*. Horm. Metab. Res..

[49] Wermuth CG (2006). Selective optimization of side activities: the SOSA approach. Drug Discov. Today.

[50] Rau O (2006). Carnosic acid and carnosol, phenolic diterpene compounds of the labiate herbs rosemary and sage, are activators of the human peroxisome proliferator-activated receptor gamma. Planta Med..

[51] Heitel P, Achenbach J, Moser D, Proschak E, Merk D (2017). DrugBank screening revealed alitretinoin and bexarotene as liver X receptor modulators. Bioorg. Med. Chem. Lett..

[52] Flesch D (2017). Non-acidic farnesoid X receptor modulators. J. Med. Chem..

[53] Schmidt J (2015). NSAIDs Ibuprofen, Indometacin, and Diclofenac do not interact with Farnesoid XReceptor. Sci. Rep..

[54] Zebisch K, Voigt V, Wabitsch M, Brandsch M (2012). Protocol for effective differentiation of 3T3-L1 cells to adipocytes. Anal. Biochem..

[55] Labute P (2008). The generalized Born/volume integral implicit solvent model: estimation of the free energy of hydration using London dispersion instead of atomic surface area. J. Comput. Chem..

[56] Willson, T. M., Brown, P. J., Sternbach, D. D. & Henke, B. R. The PPARs: From Orphan Receptors to Drug Discovery. **43** (2000). 10.1021/jm990554g 10691680

[57] Lemaire G (2006). Identification of New Human Pregnane X Receptor Ligands among Pesticides Using a Stable Reporter Cell System. Toxicol. Sci..

